# QSPR modeling of selectivity at infinite dilution of ionic liquids

**DOI:** 10.1186/s13321-021-00562-8

**Published:** 2021-10-26

**Authors:** Kyrylo Klimenko, Gonçalo V. S. M. Carrera

**Affiliations:** LAQV/REQUIMTE, Departamento de Química, Faculdade de Ciências E Tecnologia, Universidade Nova de Lisboa, Caparica, 2829-516 Caparica, Portugal

**Keywords:** Big data, Liquid mixtures, Separation technology, Keras

## Abstract

The intelligent choice of extractants and entrainers can improve current mixture separation techniques allowing better efficiency and sustainability of chemical processes that are both used in industry and laboratory practice. The most promising approach is a straightforward comparison of selectivity at infinite dilution between potential candidates. However, selectivity at infinite dilution values are rarely available for most compounds so a theoretical estimation is highly desired. In this study, we suggest a Quantitative Structure–Property Relationship (QSPR) approach to the modelling of the selectivity at infinite dilution of ionic liquids. Additionally, auxiliary models were developed to overcome the potential bias from big activity coefficient at infinite dilution from the solute. Data from SelinfDB database was used as training and internal validation sets in QSPR model development. External validation was done with the data from literature. The selection of the best models was done using decision functions that aim to diminish bias in prediction of the data points associated with the underrepresented ionic liquids or extreme temperatures. The best models were used for the virtual screening for potential azeotrope breakers of aniline + *n*-dodecane mixture. The subject of screening was a combinatorial library of ionic liquids, created based on the previously unused combinations of cations and anions from SelinfDB and the test set extractants. Both selectivity at infinite dilution and auxiliary models show good performance in the validation. Our models’ predictions were compared to the ones of the COSMO-RS, where applicable, displaying smaller prediction error. The best ionic liquid to extract aniline from *n*-dodecane was suggested.

**Supplementary Information:**

The online version contains supplementary material available at 10.1186/s13321-021-00562-8.

## Introduction

The separation of liquid mixture components is important both in industry and laboratory processes [1]. The most common separation method is distillation, however it cannot be applied to azeotropes or compounds that decompose at higher temperatures. Extractive distillation can be a good choice in case of azeotrope mixtures [2]. Thermally unstable compounds can be separated through liquid–liquid extraction [3]. Both extraction and extractive distillation require a chemical to act as an extractant/entrainer. The choice of the extractant is very important, however there are limited options for an intelligent selection with no prior experimental knowledge and it is mostly based on the comparison of the dipole moments of the solute, raffinate and extractant [4]. The intelligent entrainer choice for the breaking of the two-component mixture is usually based on the selectivity at infinite dilution ($S_{\infty }$) value for the entrainer [5]. $S_{\infty }$ value is calculated from infinite dilution activity coefficients (IDACs) that are determined via gas chromatography [6]:

1 $$ln\gamma_{1,z}^{\infty } = ln\left( {\frac{{n_{z} RT}}{{V_{n} P_{1}^{0} }}} \right) - P_{1}^{0} \times \frac{{B_{11} - V_{1}^{0} }}{RT} + \frac{{2B_{13} - V_{1}^{\infty } }}{RT} \times J \times P_{0} ,$$

where $\gamma_{1,z}^{\infty }$ is the IDAC of compound (1) in the solvent (z), $n_{z}$ is the mole number of the solvent stationary phase component inside the column, $R$ is the ideal gas constant, $T$ denotes the temperature of the oven, $V_{n}$ is the net retention volume, $B_{11}$ refers to the second virial coefficient of the solute in the gaseous state at temperature $T$. The molar volume of the solute is denoted by $V_{1}^{0}$, $B_{13}$ represents the mutual virial coefficient between the solute 1 and the carrier gas helium (index 3), $V_{1}^{\infty }$ represents the partial molar volume of the solute at infinite dilution in the solvent (extracting agent) and $P_{1}^{0}$ is the probe vapor pressure at temperature $T$. The factor $J$ amends for the influence of the pressure drop alongside the column. $P_{0}$ is the outlet column pressure. The formula is applied to calculate IDAC for both solute and raffinate and derive $S_{\infty }$ for the system at a defined pressure and temperature:

2 $$S_{\infty }^{12} = \frac{{\gamma_{\infty }^{2} }}{{\gamma_{\infty }^{1} }},$$

where $\gamma_{\infty }$ are the IDACs of a solute (1) and a raffinate (2) in the solvent. If $S_{\infty }^{12}$ $\gg$ 1, then the solvent is a good potential entrainer. There are certain limitations of this approach. For example, if $S_{\infty }^{12}$ is a result of huge $\gamma_{\infty }^{2}$ being divided by still relatively high $\gamma_{\infty }^{1}$, this would indicate that the solvent cannot separate the components, even though the $S_{\infty }^{12}$ will still be $\gg$ 1.

We have previously created the online database for the selectivities at infinite dilution and illustrated that $S_{\infty }$ can, to some extent, be an indicator of the selectivity in liquid–liquid extraction as well [7]. Database contains approximately 250 extracting solvents for two-component systems that are comprised of 154 unique chemicals at different temperatures, mounting up to 1.6 million log_10_[$S_{\infty }$] values. The solute, raffinates and solvents in the database belong to different chemical classes and the log_10_[$S_{\infty }$] values vary considerably, however there are still an enormous data gaps because of the absent experimental data on certain combinations of the above-mentioned components. While adding new experimental data to the database is possible, the data size at certain point might exceed any hosting capacity. Moreover, this data can be compiled only if someone carries out an experiment to determine the IDACs for both mixture component for the same solvent at the same temperature. This might consume less time and resources, than the direct vapor–liquid or liquid–liquid equilibria approach to determine the respected selectivity, however a considerable amount of experimental work is still required for this task. The viable alternative would be to use computational methods for the prediction of the $S_{\infty }$.

The common approach for the $S_{\infty }$ prediction is predicting the IDAC for the solute and raffinate at the same temperature and pressure separately and use those values to derive $S_{\infty }$. There have been numerous approaches for $S_{\infty }$ prediction for the liquid solutes based on very different principles, such as solvation models (SM) [8, 9], group contribution method (GCM) [10, 11], Conductor-like Screening Model for Real Solvents (COSMO-RS) [12] and Quantitative Structure–Activity Relationship (QSPR) [13–16]. SM and GCM require prior knowledge of experimental and thermodynamic parameters of solute and solvent (e.g. dispersion constant, molar volume of the solvent), which makes them less applicable to the in silico design of the extractants. COSMO-RS is an ab initio method that calculates chemical potentials, which can be used to predict the value of IDAC, so it does not require prior knowledge of the thermodynamic/experimental constants. COSMO-RS might be the most commonly used model for IDAC prediction at the moment. It allows to somewhat interpret the interactions between solvent and solute, however the model itself is quite complicated to use. In fact, its application usually requires some quantum chemical expertise and experience. Moreover, COSMO-RS is a commercial software which limits its use. QSPR models do not require any information about the compounds, apart from their chemical structures, that are used to compute independent variables known as molecular descriptors. QSPR models are purely data-driven and can be used to predict large quantity of data rather fast. There have been several attempts to make QSPR models for IDAC prediction, in most cases they were strictly local, either being restricted to a certain solute (e.g. water [14]) or a certain type of solvent (e.g. Ionic liquids (ILs) with the imidazole-based cation [15]). However, there were two attempts to make global QSPR models that can predict IDAC for various combination of solvent and solutes. In the first approach [16], a deep artificial neural network is trained on 215 ILs that act as solvents and 112 solutes. The output of the model is an IDAC prediction that fills the data gaps in solute vs. solvent data matrix. This type of output has an implicit Applicability Domain (AD) restriction, making it impossible to predict any solute or solvent if it was not in the training set. The authors claim that their dataset ‘…*represent most of known IL cation/anion and conventional solute families’*, however there might be new types of solutes/solvents discovered in the future. Moreover, the variation of the side chain in the cation allows to easily create new homologous ILs that could not be predicted by the model, although QSPR as a method does not have such restriction. The second study [13] describes the  use of  3 machine-learning methods (multiple linear regression, artificial neural network, support vector machine) to model even larger IDAC data. The models have good performance statistics, however there are several concerning issues, such as the fact that some molecular descriptors seem to be based on experimental data (e.g. dipolarity–polarizability obtained based on gas–liquid chromatography retention data on polar stationary phases). Internal validation data is used for the model parameters optimization (early stopping for ANN and kernel parameter σ2 for SVM) and small test set size (10%). Also, AD is not defined for the models at all, thus making it impossible to understand what solutes or solvents would be too different from the training data to be reliably predicted by any model. The fact that 2 IDACs bear 2 prediction errors, which can make the final prediction even more erroneous, is another issue that might occur when trying to use IDAC predictions to calculate $S_{\infty }$. To the best of our knowledge, $S_{\infty }$ has never been directly modeled using QSPR approach before. Regardless of whether $S_{\infty }$ is predicted directly or from IDACs, there is another pitfall in using the results to choose the best extractants or entrainers. It was discussed in our previous article [7], as a big IDAC issue, i.e. a situation when a high $S_{\infty }$ value is achieved by dividing very big IDAC by a smaller, yet big IDAC. The common chemistry knowledge indicates that in this case the separation of two-component mixture by the examined solvent is impossible and big IDAC issue has to be taken into account when the decision on extractant selection is made.

ILs seem to be the focus solvents when it comes to QSPR modeling of the IDAC. The reason for this lies in IL’s ‘sustainable’ properties such as low flammability hazard, [17] negligible vapour pressure at standard working conditions, [18] and moderate viscosities [19]. IL’s simultaneously have high structural variability and consist of well-defined types of ions. This creates opportunities for intelligent molecule design and should be a good working framework for QSPR approach, that relies on determination of structure–property patterns.

The modelling of $S_{\infty }$ for ILs is not a trivial QSPR problem. In classic QSPR, one data point corresponds to one chemical. The $S_{\infty }$ value for every data point is determined by the cation, anion, solute, raffinate and temperature ($T$), with the atmospheric pressure implied in this case. This makes the task at hand a modelling of a mixture property of 4 components with a varying non-structural parameter ($T$). Mixture and parameter-dependent properties pose additional modelling challenges, such as molecular descriptor choice [20], test set selection [21–23], error estimation [22], model sensitivity to the parameter impact [24] and AD definition [22]. Most machine-learning algorithms tend to optimize the model around successful average predictions, so the model might end up only predicting well data points associated with the most common IL, solute, $T$ or other parameters. An appropriate model optimization must be done in order to avoid that.

In this study, the QSPR model for predicting log_10_[S_∞_] for ILs was developed and additional big IDAC classification model was done to flag unreliable results. The developed models were used to predict the best possible breakers of aniline + dodecane azeotrope.

## Materials and methods

### Data standardization and curation

SelinfDB [7] is the source of data for model development and internal validation. The data comprises names of cations, anions, solutes and raffinates, as well as, temperature, log_10_[S_∞_] and bigIDAC flag. The decimal logarithm is important for scaling the property to the orders of magnitude that reflect the extracting potential better, than the absolute values. The SMILES format chemical structure representation of cation and anion was taken from Additional file 1: Table S1 Electronic supplementary information (ESI) of Paduszyński [25]. This type of representation for solutes and raffinates was generated with OPSIN software [26] from components names. Next, the non-IL data was removed from the dataset. Structure representation was standardized using Chemaxon Standardizer [27]: standardization rules are described in [22].

### QSPR model development

Then, molecular descriptors were generated separately for each component (cation, anion, solute, raffinate) using Chemaxon cxcalc plug-in [28]. Molecular descriptors chosen in this study reflect components physico-chemical nature (e.g. logP, Polar surface area) and structural features (e.g. number of rotatable bonds, number of aromatic atoms). Full list of molecular descriptors can be found in Additional file 1: Table S1.

The QSPR model development was done using Artificial Neural Network (ANN) machine-learning method from Keras in R language environment [29–31] for both log_10_[S_∞_] and bigIDAC flag property. Models developed in this study have both fixed and tunable parameters, the latter required for assuring the best performance. The fixed parameters are:

- Number of hidden layers (2)
- Activation functions (parametric relu and relu for hidden layers. For output layers, sigmoid in case of log_10_[S_∞_] or linear for bigIDAC flag modelling)
- Metric for error assessment (mean absolute error for log_10_[S_∞_] and binary cross-entropy for and bigIDAC flag)
- Batch size (2000)
- Number of iteration cycles (500)

The tunable parameters are:

- Weight initializer (glorot, lecun and he options for generating values based on uniform distribution)
- Number of nodes in hidden layers (100 and 200 for the first hidden layer; 10, 20, 40 for the second one)
- Optimization function (rmsprop, adamax, adadelta, sgd)

The in-built keras optimization finds the most optimal values for the weights in layers, however it does not affect tunable parameters. The best option for the tunable parameters were determined using exhaustive search. The criterion for the best option was model’s predictivity of the optimization set. The optimization set was formed by setting aside the part of the dataset and not using it during model development. The optimization set must reflect the structural diversity of the data and also challenge the model algorithm, however it should not contain chemicals that are too structurally different from the training set, since that will make successful predictions impossible. Two steps were taken in order to assure a good tradeoff between having familiar and unfamiliar patterns in the optimization set. Firstly, the data points selection was done on the IL-out basis, i.e. all data points from the same IL must be present either in the training or in the optimization set. However, the cation or anion from the IL may be a part of both sets. Secondly, the IL selection was done based on the Euclidean distance between ILs in the descriptor space, similar to the procedure described in [22]. The selection was done as follows: the Euclidean distance in the descriptor space for all ILs is calculated using SARA software [32, 33]. Then, median distance is found for every IL. Next, the median distance of the median distances is found and used as a threshold (Eq. 3). Finally, the first 10% of ILs that had median values higher than the threshold and the first 10% of ILs that had median values lower than the threshold are selected for the optimization set.

3 $$Thr = \mathop {{\text{median}}}\limits_{j = 1} \left( {\mathop {{\text{median}}}\limits_{i = 1} \left( {d_{ij} } \right)} \right),$$

where *Thr* is a threshold value, $d_{ij}$ is Euclidean distance between the $i$-th compound to the $j$-th compound in the data subset ($i = j$ = *number of compounds in the subset*).

The assessment of models predictivity in this case is challenging due to dataset size and the fact that it’s a mixture property, thus decision functions were used to select the best model for both properties. In this study, decision functions are geometric means of other metrics. The log_10_[S_∞_] metrics include Mean Absolute Error ($MAE$) for overall prediction accuracy, Averaged Mean Absolute Error per IL ($MAE_{IL}$) for prediction accuracy across extractants, difference between observed and predicted covariances of property and temperature ($\Delta Cov\left( {{\text{log}}_{10} \left[ {{\text{S}}_{\infty } } \right],{\text{ T }}} \right)$) to assure that models reflect temperature-dependency of the property, the difference between the ranges of observed and predicted values ($\Delta range$) to assure that model does not simply average all predictions and mispredict extreme values:

4 $$MAE = \frac{{\mathop \sum \nolimits_{i = 1}^{n} \left| {y_{i} - x_{i} } \right|}}{n},$$

where $n$ is the number of data points, $y_{i}$ is predicted values and $x_{i}$ is observed values for the $i$-th data point

5 $$MAE_{IL} = \frac{1}{k}\left( {\mathop \sum \limits_{j = 1}^{k} \frac{{\mathop \sum \nolimits_{i = 1}^{n} \left| {y_{ji} - x_{ji} } \right|}}{n}} \right),$$

where $k$ is the number of compounds in the optimization set, $n$ is the number of data points per compound, $x_{ji}$ is the experimental values for the $j$-th compound. $y_{ji}$ is the predicted values for the $j$-th IL for the $i$-th data point.

6 $$\Delta Cov\left( {\log_{10} \left[ {{\text{S}}_{\infty } } \right],\; {\text{T}} } \right) = \left| {\frac{1}{n}\sum \left( {x_{i} - \overline{x}} \right)\left( {T_{i} - \overline{T}} \right) - \frac{1}{n}\sum \left( {y_{i} - \overline{y}} \right)\left( {T_{i} - \overline{T}} \right) } \right|,$$

where $n$ is the number of data points, $y_{i}$ is predicted values and $x_{i}$ is observed values, $\overline{x}$ is average observed value, $\overline{y}$ is average predicted value, $T_{i}$ is temperature of $i$-th data point, $\overline{T}$ is the average temperature.

7 $$\Delta range = \left| {\left( {\max \;x_{i} - \min \;x_{i} } \right) - \left( {\max \;y_{i} - \min \;y_{i} } \right)} \right|.$$

The decision function for log_10_[S_∞_] is as follows:

8 $$Df_{{{\text{log}}10\left[ {{\text{S}}_{\infty } } \right]{ }}} = \sqrt[4]{{MAE \times MAE_{IL} \times \Delta Cov\left( {{\text{log}}_{10} \left[ {{\text{S}}_{\infty } } \right],\;{\text{T }}} \right) \times \Delta range}}.$$

The metrics used for bigIDAC flag model performance assessment included Balance Accuracy ($BA$) to assure correct predictions of both normal and problematic data points and Accuracy per IL ($Acc_{IL}$) for the reasons mentioned in $MAE_{IL}$ description:

9 $$BA = {{\left( {\frac{TP}{{TP + FN}} - \frac{TN}{{TN + FP}}} \right)} \mathord{\left/ {\vphantom {{\left( {\frac{TP}{{TP + FN}} - \frac{TN}{{TN + FP}}} \right)} 2}} \right. \kern-\nulldelimiterspace} 2} ,$$

where $TP$ is data points that have big IDAC flag that are predicted to have big IDAC flag, $TN$ is data points that do not have big IDAC flag that are predicted not to have big IDAC flag, $FN$ is data points that have big IDAC flag that are predicted not to have big IDAC flag, $FP$ is data points that do not have big IDAC flag that are predicted to have big IDAC flag. $Acc_{IL}$ formula is similar to the $MAE_{IL}$ one, only applied to bigIDAC flag binary data.

The decision function for bigIDAC flag is as follows:

10 $$Df_{{{\text{bigIDAC}}\;{\text{flag}} }} = \sqrt[2]{{BA \times Acc_{IL} }}.$$

The geometric mean approach was used to ensure that every metric performance is contributing equally to the decision function value. Best models are the ones with the lowest and highest decision function value for log_10_[S_∞_] and bigIDAC flag optimisation set predictions, respectively.

### Cross-validation scheme examination

The secondary objective of this study was to examine the influence of the internal validation set size on model’s predictivity. The examination was done by building models with 20, 50 and 80% of the QSPR-ready SelinfDB data in the validation set as a part of the fivefold random cross-validation procedure, ensuring that every data point will be present in the validation set at least one time. The 5 models developed for the respective folds were used to predict optimization set with the final result being an averaged prediction for each data point. The selection of the optimized parameters was done in case of every CV split approach. The input molecular descriptors for the training + internal validation sets were scaled using linear scaling with variable range [34], making every descriptor value reside within 0–1 limits. The rounding of the scaled descriptors was done up to the first digit after the decimal point. The derived linear coefficient and free term were used to scale the optimization set. The Applicability Domain (AD) was determined using the Bounding box method: a p-dimensional hyper-rectangle defined on the basis of maximum and minimum values of each descriptor used to build the model [35]. In other words, every descriptor value of examined compound must be within the range of the corresponding descriptor from the training set. The AD definition was applied to the unscaled descriptors, i.e. a data point is considered to be within AD if each of its descriptor value is within the training set value range of a respective descriptor.

### External validation

Models predictivity was assessed using external test set. This set was composed from the IDAC data for ILs found in literature between years 2018 and 2020. The list of publications is given in Additional file 1: Table S2. The calculation of log_10_[S_∞_], duplicate removal and other data processing was done as described in [7]. Molecular descriptors for known components, i.e. cations, anions, solutes and raffinates, that were present in SelinfDB, were copied from the training and optimization set; descriptors for new components were calculated using cxcalc plugin in the same manner as it was for the training and optimization set. Descriptor scaling, averaging prediction for the final result and determination of the AD for the external test set was done in the same manner as it was for the optimization set, however only the optimal models from every CV split were used for external test set predictions. The external test set predictivity was used as a criterion for choosing the best of the best models for both log_10_[S_∞_] and bigIDAC flag. These models were used to demonstrate the potential of QSPR approach by predicting the azeotrope-breaking potential of ILs from computational combinatorial library on separation of the aniline + dodecane mixture at 298 K. The computational combinatorial library was created by generating all possible combinations of cations and anions present in the SelinfDB and external test set and then removing the ILs that have been previously used. An azeotrope containing aniline [36] was chosen because aniline has not been present in a previously used datasets and can show the potential of the models to give deal with novel compounds.

### Virtual screening

Additionally, the log_10_[S_∞_] prediction results for the optimization set were compared to the COSMO-RS predictions from [25] COSMO-RS log_10_[S_∞_] was created by choosing predicted IDAC data from column *IDAC (calcd)* of Table 4 in ESI of Paduszyński article [25]. Then, log_10_[S_∞_] was calculated in the same manner as SelinfDB data.

**Fig. 1:**
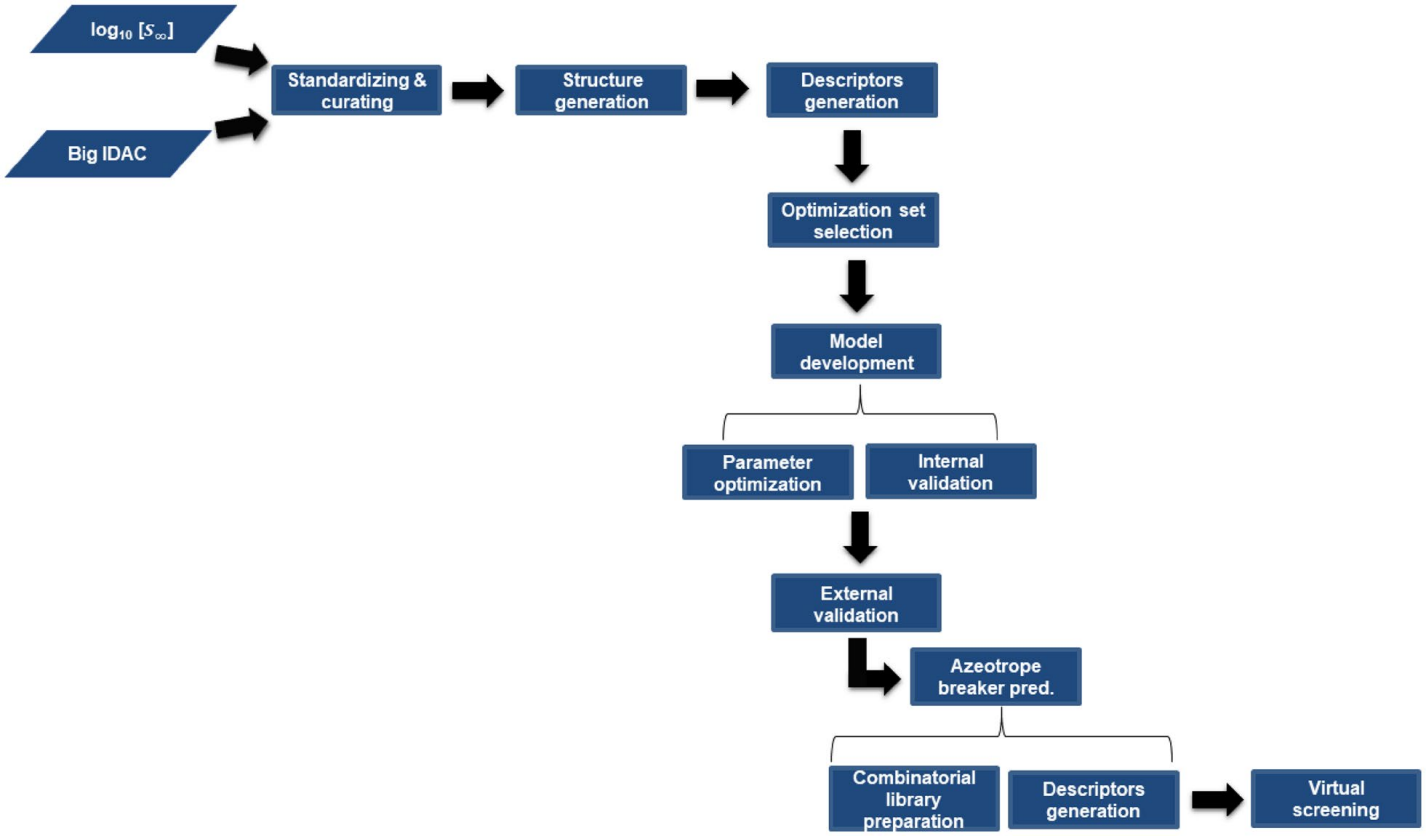
Workflow scheme

A Linux (Centos 6) cluster with SLURM was used for the ANN development, optimization and external test set prediction, as well as prediction of aniline + dodecane breakers. The nodes were Intel Xeon E5-2630 CPUs and NVIDIA GeForce GTX TITAN X GPUs. NVIDIA CUDA libraries, that were needed for running keras and tensorflow, are version 10.1 (Fig. 1).

## Results and discussion

### Data standardization, curation and model development

The IL data from SelinfDB has 1,614,570 data points, describing 226 extracting solvents for two-component systems. The number of data points was reduced to 1,413,780 after verification of some structure-descriptor calculation failures. The median of the median of the median optimization set selection resulted in 308,433 data points (42 ILs) set aside for the optimisation set. The results of the optimization are given in Tables 1 and 2.

**Table 1:** Optimization set prediction statistics for log_10_[S_∞_]

	Split (CV. %)	MAE	MAE per IL	Range	Covariance	Decision
log_10_[S_∞_]	20	0.119	0.170	0.24	5.23E−06	0.0126317
	50	0.128	0.173	0.01	0.00014608	0.0134264
	80	0.132	0.177	0.66	3.83E−06	0.0155686

**Table 2:** Optimization set prediction statistics for bigIDAC flag

	Split (CV. %)	Sensitivity	Specificity	BA	Acc	Acc (per IL)	Decision
bigIDAC	20	0.868	0.956	0.912	0.929	0.944	0.928
	50	0.845	0.965	0.905	0.928	0.941	0.923
	80	0.869	0.950	0.909	0.925	0.934	0.922

All of the optimization set data points were within AD. The ANN tunable parameter values for the best log_10_[S_∞_] and bigIDAC models are given in Additional file 1: Table S3. The results show good predictivity for both log_10_[S_∞_] and BigIDAC models. The ‘per IL’ statistics are worse in terms of absolute error for log_10_[S_∞_] models—this has been observed in previous QSPR studies of equilibria-based properties [22] and is concordant with the fact that some systems tend to be predicted less accurately due to noise in experimental data or unusual behaviour [21].

### Models’ validation

The results of the best models for log_10_[S_∞_] were compared to the results from COSMO-RS. Due to the duplicate removal procedure described in [7], the COSMO-RS log_10_[S_∞_] values could not be calculated for all data points in the optimization set, however they have 99.85% overlap, which we believe is sufficient for the comparison. COSMO-RS prediction had the following statistics: MAE of 0.203, MAE per comp of 0.272, range difference of 1.64, covariance difference of 0.029207799 and a decision function of 0.226805763. This is significantly less accurate, than our predictions.

After the best models from every CV approach were selected, they were used to predict the external test set that has 511,496 data points: 42 ILs, 6778 mixtures retrieved from 28 articles. The prediction results are given in Tables 3, 4, and 5.

**Table 3:** Test set prediction statistics for log_10_[S_∞_]

	Split (CV. %)	MAE	MAE per IL	Range	Covariance	Decision
log_10_[S_∞_]	20	0.180	0.204	1.46	0.000377	0.066979
	50	0.179	0.205	1.1	0.001127	0.08218
	80	0.164	0.190	0.49	0.002173	0.075874

**Table 4:** Categorical interpretation of test set prediction statistics for log_10_[S_∞_]

	Split (CV. %)	Acc	Sensitivity	Specificity	BA	PPV	NPV
log_10_[S∞]	20	0.962	0.887	0.974	0.930	0.839	0.983
	50	0.962	0.848	0.979	0.914	0.862	0.977
	80	0.968	0.872	0.983	0.927	0.886	0.980

**Table 5:** Test set prediction statistics for bigIDAC flag

	Split (CV. %)	Sensitivity	Specificity	BA	Acc	Acc (per IL)	Decision
BigIDAC	20	0.811	0.978	0.895	0.934	0.921	0.908
	50	0.813	0.978	0.895	0.934	0.923	0.909
	80	0.802	0.985	0.894	0.937	0.924	0.909

All of the test set data points were within AD. Test set predictions are worse, than the optimization ones, both in terms of error (e.g. MAE, MAPE, BA) and decision function, with the exception of Accuracy for bigIDAC models. Nonetheless, models’ performance in the external validation is quite good, including the classification interpretation of log_10_[S_∞_] results.

Test set data for main and auxiliary properties have shown better results for having less data points in the CV set, which is contrary to the optimization results. The test set evaluation is a better approach for the assessment of predictive ability than optimization set, however this fluctuation can be attributed to the chance due to randomization before CV split or random number generation for the initial weights generation. In order to examine this possibility, 4 more models with optimized hyperparameters were generated for 80% CV split and their performance on the external test set was averaged. Confidence interval (Eq. 11) must be calculated to test whether 20% CV split test prediction result can be achieved by random deviation from 80% CV split test set results:

11 $$CI = \overline{MAE} \pm z_{0.95} \times \frac{{\sigma \left( {MAE} \right)}}{\sqrt n },$$

where $CI$ is a confidence interval, $\overline{MAE}$ is an average MAE of 5 80% CV split models, $z_{0.95}$ is a Z-score of 95% confidence level, $\sigma \left( {MAE} \right)$ is a standard deviation of MAE of 5 80% CV split models, n is the number of optimized models. The calculation result is $0.164 \pm 1.96 \times \frac{{0.002{ }}}{\sqrt 5 } = \left[ {0.162;0.166} \right]$ $CI$. The 20% CV result is out of the CI, so the difference in performance is less likely to be achieved randomly.

**Fig. 2:**
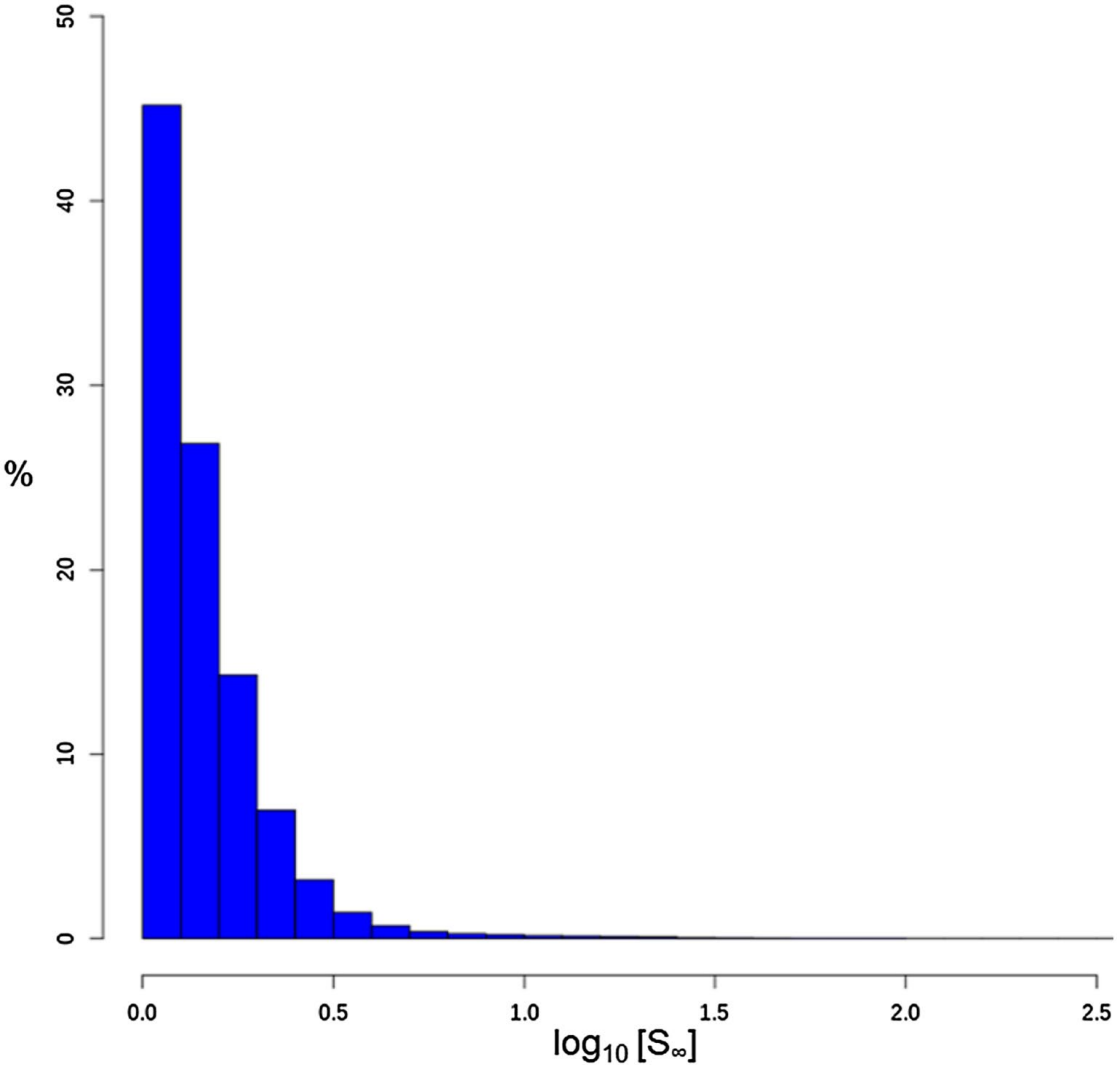
Absolute error distribution (X-axis) in the external test set predicted by the best log_10_[S_∞_] model

The efficiency of the final model’s ability to predict log_10_[S_∞_] can be further illustrated by the distribution of the absolute error (Fig. 2). It is clear that more than 90% of data points from the external test set have a prediction error of less than 0.5 log_10_[S_∞_], i.e. half of the order of magnitude for S_∞_. The results for the training and internal validation are given in Table 6.

**Table 6:** Training and internal validation statistics for the best of the best log_10_[S_∞_] and BigIDAC models

	Mean ± sd
log_10_[S_∞_]	
MAE (TrS)	0.11 ± 0.02
MAE (Val)	0.12 ± 0.02
BigIDAC	
BinCross (TrS)	0.01522 ± 0.00222
BinCross (Val)	0.02479 ± 0.00297
Acc (Val)	0.98895 ± 0.00063

### Virtual screening

The (computational) combinatorial library of ILs was created using all possible combinations of cations and anions from the SelinfDB and external test set. Only 249 out of 5200 (4.8%) combinations were experimentally tested. The combinations that were previously explored were discarded. The rest (4951 ILs) have been used to predict the separation of aniline form *n*-dodecane acetate. Full information on explored and unexplored combinations is given in Additional file 2.

log_10_[S_∞_] prediction values vary from 0.03 to 3.96. No ILs had a bigIDAC warning label assigned. All predictions were within AD. The majority of most promising ILs (Table 7) contain 4-(3-hydroxypropyl)-4-methylmorpholinium (MO-3OH,1) cation. All cations from top 10 results contain hydroxy (OH) or methoxy (O1) groups. The translation of the codes can be found at https://selinfdil.dq.fct.unl.pt/il-codes-translation/.

**Table 7:** Top 10 ILs suggested for breaking of aniline + *n*-dodecane azeotrope

ILs	log_10_[$S_{\infty }$]
MO-3OH,1_AC	3.96
MO-3OH,1_BF4	3.95
MO-3OH,1_CL	3.95
MO-3OH,1_SCN	3.93
MO-3OH,1_BR	3.92
[(OH)2C3Mpyr]_BF4	3.89
PYR-2OH,1_AC	3.87
MO-3OH,1_PO3H-2	3.86
PYR-2OH,1_SCN	3.83
IM-O1,O1_CL	3.81

**Fig. 3:**
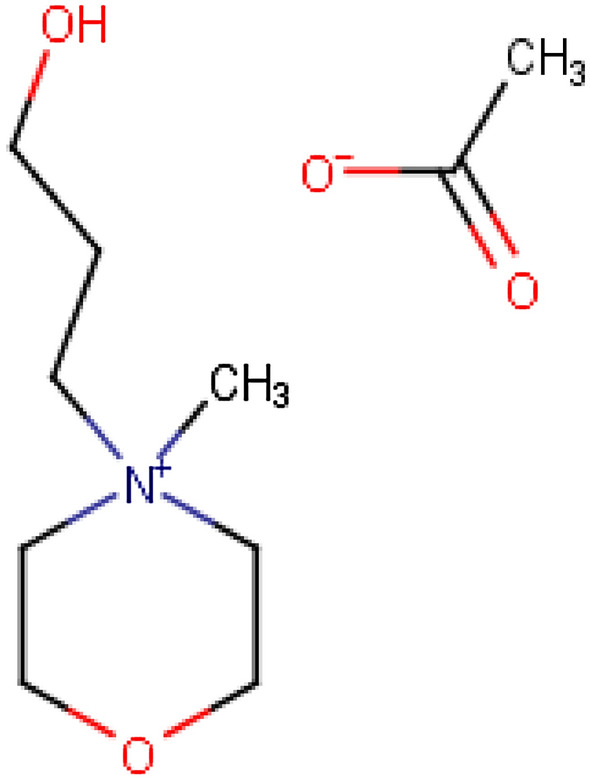
IL suggested for breaking aniline + *n*-dodecane azeotrope

The best candidate for breaking aniline + *n*-dodecane azeotrope is 4-(3-hydroxypropyl)-4-methylmorpholinium acetate (Fig. 3). To the best of our knowledge, this IL has never been experimentally tested for the separation of aniline from *n*-dodecane, or any other separation process.

## Conclusions

QSPR models for log_10_[S_∞_] and BigIDAC flag developed in this study are rather precise and can be used to predict the extractive potential for unexplored combinations of cation/anion/solute/raffinate/temperature, even if they were not present in the original dataset. Several ILs are suggested for the breaking of aniline/*n*-dodecane azeotrope. ANN method has been successful in modeling with repetitive patterns, such as temperature impact and small structure variability of mixture property. The use of integral molecular descriptors, rather than fragment ones, resulting in lower descriptor space dimensionality, was probably the right choice for the modeling of this dataset as well. The increase in the CV fold size led to better predictivity of the models in general, possibly due to diminished overfit from above-mentioned repetitive data being used less in ANN model development.

## Supplementary Information

**Additional file 1: Table S1.** List of molecular descriptors used in the study. Temperature was an additional non-molecular parameter. In total 69 independent variables were used in ANN model development. **Table S2.** Literature sources used for the test set formation. **Table S3.** Best models optimized parameter values.

**Additional file 2. **Explored and unexplored combinations of cations and anions for the computational combinatorial library of ILs are given in the combinatorial_library.txt file, where rows describe cations, columns describe anions, ‘X’ means that the combination has already been explored in either training, optimization or test set and ‘O’ means that the combination has not been explored.

## Data Availability

The data files and scripts needed to reproduce the results of the QSPR modeling are given in https://github.com/klimenko-od91/QSPR_Selinfdil_ILs repository.
