# Supplementary material for: QSPR modeling of selectivity at infinite dilution of ionic liquids
Source: J Cheminform. 2021 Oct 26;13:83. doi: 10.1186/s13321-021-00562-8 (PMC8549394; doi:10.1186/s13321-021-00562-8)
Supplement: Supplementary file 1 — Additional file 1: Table S1. List of molecular descriptors used in the study. Temperature was an additional non-molecular parameter. In total 69 independent variables were used in ANN model development. Table S2. Literature sources used for the test set formation. Table S3. Best models optimized parameter values. [file 13321_2021_562_MOESM1_ESM.docx]

| **These descriptors were calculated for every component (cation, anion, solute, raffinate). 68 molecular descriptors in total** |
| --- |
| logP |
| Atom count |
| Molecular weight |
| Average polarizability (molecular) |
| Aliphatic atom count |
| Aromatic atom count |
| Polar surface area |
| donorcount |
| acceptorcount |
| Refractivity |
| Bond count |
| Fused ring count |
| Chain atom count |
| Balaban index |
| Rotatable bond count |
| Smallest ring size |
| Ring count |

Table S1 List of molecular descriptors used in the study. Temperature was an additional non-molecular parameter. In total 69 independent variables were used in ANN model development.

| 1. | Mutelet F, Ravula S, Baker GA et al. (2018) infinite dilution activity coefficients and gas-to-liquid partition coefficients of organic solutes dissolved in 1-benzylpyridinium bis(trifluoromethylsulfonyl)imide and 1-cyclohexylmethyl-1-methylpyrrolidinium bis(trifluoromethylsulfonyl)imide. J Solution Chem, 47:308–335 |
| --- | --- |
| 2. | Mutelet F, Baker GA, Ravula S et al. (2019) Infinite dilution activity coefficients and gas-to-liquid partition coefficients of organic solutes dissolved in 1-sec-butyl-3-methylimidazolium bis(trifluoromethylsulfonyl)imide and in 1-tert-butyl-3-methylimidazolium bis(trifluoromethylsulfonyl)imide. Phys Chem Liq, 57,4: 453-472 |
| 3. | Kabane B, Arumugam V, Chokkareddy R, Redhi GG (2019) Assessment of pyrrolidinium-based ionic liquid for the separation of binary mixtures based on activity coefficients at infinite dilution. J Chem Eng Data, 64,12:5105-5112 |
| 4. | Domańska U, Wlazło M, Karpińska M (2020) [DCA]-based ionic liquids for the extraction of sulfur and nitrogen compounds from fuels: Activity coefficients at infinite dilution. Fluid Phase Equilib, 507:112424 |
| 5. | Kabane B, Redhi GG (2019) Application of trihexyltetradecylphosphonium dicyanamide ionic liquid for various types of separations problems: Activity coefficients at infinite dilution measurements utilizing GLC method. Fluid Phase Equilib, 493:181-187 |
| 6. | Ziemblińska-Bernart J, Bielecki P, Wasiak W (2019) Measurements of activity coefficients at infinite dilution for organic solutes in two quaternary ammonium-based ionic liquids [DDA][ClO4] and [DDA][BF4]. Fluid Phase Equilib, 482:99-107 |
| 7. | Domańska U, Karpińska M, Wiśniewska A, Dąbrowski Z (2019) Ammonium ionic liquids in extraction of bio-butan-1-ol from water phase using activity coefficients at infinite dilution. Fluid Phase Equilib, 479:9-16 |
| 8. | Domańska U, Wlazło M, Karpińska M, Zawadzki M (2018) New ionic liquid [P4,4,4,4][NTf2] in bio-butanol extraction on investigation of limiting activity coefficients. Fluid Phase Equilib, 475:89-94 |
| 9. | Durski M, Naidoo P, Ramjugernath D, Domańska U (2018) Thermodynamics and activity coefficients at infinite dilution for organic solutes in the ionic liquid 1-butyl-1-methylpyrrolidinium dicyanamide. Fluid Phase Equilib, 473:175-182 |
| 10. | Karpińska M, Wlazło M (2020) Application of dicyanamide-based ionic liquid in separation of binary mixtures based on gamma infinity data measurements. J Mol Liq, 310:113176 |
| 11. | Rabhi F, Hussard C, Sifaoui H, Mutelet F (2019) Characterization of bis(fluorosulfonyl)imide based ionic liquids by gas chromatography. J Mol Liq, 289: 111169 |
| 12. | Rabhi F, Mutelet F, Sifaoui H et al. (2019) Characterization of the solubilizing ability of tetraalkylammonium ionic liquids containing a pendant alkyl chain bearing a basic N,N-dimethylamino or N,N-dimethylaminoethoxy functionality. J Mol Liq, 283:380-390 |
| 13. | Paduszyński K, Królikowski M, Orzeł P (2019) Thermodynamic properties of infinitely diluted solutions of organic solutes in in silico designed task-specific ionic liquid. J Mol Liq, 279:733-739 |
| 14. | Zhang R, Bao YN, Zhang L, Ren RZ, Yu-Hai Jiao YH, Ge ML (2020) Thermodynamics and selectivity of separation based on activity  coefficients at infinite dilution of various solutes in ionic liquid [DMIM]  [Tf2N]. J Chem Therm, 147:106120 |
| 15. | Wang W, Wang Q, Tang J et al. (2020) Characterization of the thermodynamic properties of ionic liquid 1-allyl-3-vinylimidazolium bis((trifluorompropyl)sulfonyl)imide by inverse gas chromatography. J Chem Therm, 150:106236 |
| 16. | Królikowski M, Królikowska M, Więckowskia M, Piłowski A (2020) The influence of the ionic liquids functionalization on interaction in binary systems with organic solutes and water – Thermodynamic data of activity coefficients at infinite dilution. J Chem Therm, 147:106117 |
| 17. | He ZZ, Li RQ, Sun AL, et al. (2020) Experimental and theoretical study on infinite dilution activity coefficients of various solutes in ionic liquid 1-propyl-2,3-dimethylimidazolium bis(trifluoromethylsulfonyl)imide. J Chem Therm, 140:105894 |
| 18. | Zhang C, Triger D, Nicholas NJ (2020) Activity coefficients at infinite dilution for various organic solutes in the ionic liquid 1-(2-hydroxyethyl)-3-methylimidazolium hexafluorophosphate. J Chem Therm, 140:105867 |
| 19. | Kabane B, Chokkareddy R, Redhi GG (2019) Separation of (water/butan-1-ol) binary systems based on activity coefficients at infinite dilution with phosphonium ionic liquid. J Chem Therm, 137:7-12 |
| 20. | Zhang M, Ge ML, Jiao YH et al. (2019) Determination of the thermodynamic parameters of ionic liquid 1-propyl-3-methylimidazolium bromide by gas-liquid chromatography. J Chem Therm, 129:92-98 |
| 21. | Domańska U, Karpińska M (2018) The use of ionic liquids for separation of binary hydrocarbons mixtures based on gamma infinity data measurements. J Chem Therm, 127:95-105 |
| 22. | Zhang M, He ZZ, Kang RX, Ge ML (2019) Thermodynamics and activity coefficients at infinite dilution for organic compounds in the ionic liquid 1-hexyl-3-methylimidazolium chloride. J Chem Therm, 128:187-194 |
| 23. | He ZZ, Zhai JY, Mu H et al. (2018) Thermodynamics and selectivity of separation based on activity coefficients at infinite dilution of various solutes in ionic liquid [HMMIM][BF4]. J Chem Therm, 125: 142–148 |
| 24. | Marciniak A, Wlazło M (2018) Activity coefficients at infinite dilution and physicochemical properties for organic solutes and water in the ionic liquid trihexyl-tetradecylphosphonium tricyanomethanide. J Chem Therm, 120:72-78 |
| 25. | Chen JY, Kang RX, He ZZ et al. (2018) Separation of binary mixtures based on gamma infinity data using [OMMIM][NTf2] ionic liquid and modelling of thermodynamic functions. J Chem Therm, 119:26-33 |
| 26. | Wlazło M, Zawadzki M, Domańska U (2018) Separation of water/butan-1-ol based on activity coefficients at infinite dilution in 1,3-didecyl-2-methylimidazolium dicyanamide ionic liquid. J Chem Therm, 116:316-322 |
| 27. | Mutelet F, Hussard C, Baker GA et al. (2020) Characterization of the solubilizing ability of short-chained glycol-grafted ammonium and phosphonium ionic liquids. J Mol Liq, 304: 112786 |
| 28. | Li C, Ban T, Wang Q (2020) Activity coefficient at infinite dilution study of molecular interaction‑selectivity in separation processes. Int J Thermophys, 41:122 |

Table S2 Literature sources used for the test set formation

|  |  | CV split (%) | optimization | initializer | nodes1 | nodes2 |
| --- | --- | --- | --- | --- | --- | --- |
| **log_10_[S_∞_]** |  | 20 | sgd | glorot_uniform | 200 | 10 |
|  |  | 50 | rmsprop | he_uniform | 100 | 40 |
|  |  | 80 | sgd | lecun_uniform | 200 | 10 |
|  |  |  |  |  |  |  |
| **bigIDAC** |  | 20 | rmsprop | he_uniform | 100 | 10 |
|  |  | 50 | rmsprop | glorot_uniform | 100 | 20 |
|  |  | 80 | rmsprop | he_uniform | 100 | 20 |

Table S3 Best models optimized parameter values
